# The Clinical Effect and Mechanism of Prostant on Urinary Retention and Anal Pain

**DOI:** 10.1155/2022/2570169

**Published:** 2022-09-06

**Authors:** Wei-Min Luo, Han Du, Hong-Liang Jiang, Ying-Jun Deng, Xue Liang, Ping Qiu, Yao Cheng

**Affiliations:** ^1^Department of Colorectal Surgery, The First Affiliated Hospital of Guangzhou University of Chinese Medicine, Guangzhou 510405, China; ^2^Department of Dermatology, Gaozhou Hospital of Traditional Chinese Medicine Affiliated to Guangzhou University of Chinese Medicine, Gaozhou 525025, China; ^3^Department of Anorectal Medicine, Gaozhou Hospital of Traditional Chinese Medicine Affiliated to Guangzhou University of Chinese Medicine, Gaozhou 525025, China; ^4^Graduate School of China Academy of Chinese Medical Sciences, Beijing 100091, China; ^5^Department of Prevention and Health Care, Gaozhou Hospital of Traditional Chinese Medicine, Guangzhou University of Chinese Medicine, Gaozhou 525025, China

## Abstract

Anal pain and urinary retention are the two most outstanding complications of the procedure for prolapse and hemorrhoids (PPH) surgery. This study intended to assess the clinical effect and mechanism of Prostant on urinary retention and anal pain after the PPH. Here, 30 patients received PPH surgery. The role and mechanism of Prostant in patients and mice with urinary retention and anal pain were evaluated. ANOVA tests were executed and differences between groups were regarded as statistically significant when *p* < 0.05. Prostant effectively improved the urination status, lower abdomen symptoms, time to urinate and score of VAS, and the reduction of TNF-*α* and IL-6. Similarly, Prostant can ameliorate the outcome of urodynamics in urinary retention mice. Mechanically, Prostant reversed the urinary retention-elevated the serum level of hs-CRP and TNF-*α*, reduction of IL-2, imbalance of Treg/Th17, and level of JAK2 and phosphorylated STAT3. Besides, Prostant ameliorated the pain as shown by the reduction of writhing response, and the elevation of threshold of pain and degree of swelling. Moreover, Prostant antagonized the pain-induced dysregulation of Treg/Th17. Therefore, Prostant can treat patients and mice with anal pain and urinary retention by modulating the balance of Th17/Treg to regulate the secretion and production of inflammatory factors. We hope our results can establish a scientific treatment approach for solving anal pain and urinary retention after PPH surgery of mixed hemorrhoids.

## 1. Introduction

Hemorrhoids are one of the remarkable diseases manifested as the anal mucosa prominences that are generated by loose connective tissue, venous and arterial vessels, and smooth muscle [1]. In the clinic, hemorrhoids represent a variety of symptoms, including bleeding, pain, soiling, and itching, which had seriously affected patients' health and life [2]. Moreover, it has been reported that hemorrhoids in adults are prevalent all over the world, especially in elderly adults and pregnant women [2]. Although pharmacotherapy is an effective nonsurgical therapy for patients with mild hemorrhoids, patients with hemorrhoids generally develop a severe course owing to their insufficient perception and shame to seek medical treatment [1, 3]. Thus, surgical procedures have been a necessary therapy for patients with severe hemorrhoids.

The procedure for prolapse and hemorrhoids (PPH) surgery was first mentioned in 1998 by Longo, which then has been widely utilized in the therapy of hemorrhoids [4]. However, clinical studies have revealed that there is a series of complications after PPH. Therein, anal pain and urinary retention are the two most outstanding complications. The incidence of urinary retention in mixed hemorrhoids after surgery is 20% to 52% [5], while moderate to severe anal pain also occurs in more than 60% of postoperative patients [6]. Furthermore, anal pain and urinary retention promote each other to form a vicious circle, which causes severe pain to patients and hinders the healing of postoperative wounds. Therefore, it is urgently and crucial to effectively reduce the incidence of urinary retention and anal pain after hemorrhoid surgery.

“Treating different diseases with the same treatment” is an important fundamental theory of traditional Chinese medicine (TCM), which indicates that the same approach of treatment can be used to treat diverse diseases with analogical pathological alteration during various stages of the progression [7]. TCM theories believe that the pathological mechanism of complications such as urinary retention and anal pain after PPH surgery of damp-heat mixed hemorrhoids is damp-heat stasis, which is similar to the pathogenesis of the damp-heat stasis type lower scorch disease such as chronic prostatitis [8–11]. Prostant is a traditional Chinese patent medicine generally applied in China for the treatment of chronic prostatitis, which mainly comprises *Cortex Phellodendri* (Huang Bai), *Rhizoma Polygoni* Cuspidati (Hu Zhang), *Fructus Gardeniae* (Zhi Zi), and *Radix et Rhizoma Rhei* (Da Huang) [12]. The main active ingredient of Prostant is berberine isolated from Huang Bai that can clear away heat, and remove dampness and blood stasis. Modern medicine has proved that berberine can reduce the local inflammatory response, decline inflammatory stimulation to the local area, and relieve the convulsions of the bladder neck, and urethral sphincter by inhibiting the release of local inflammatory factors [13, 14]. In addition, Prostant also has antibacterial effects and regulates mental emotions that have been demonstrated the safety in clinical application [12]. Prostant takes the prostate, bladder neck, and rectum as the main targets, therefore, it should be put 3 to 4 cm inside the opening of the rectum based on the instruction. Thus, Prostant may be used to treat urinary retention and anal pain after PPH surgery of damp-heat mixed hemorrhoids following the “Treating different diseases with the same treatment” theory.

Here, the clinical effects of Prostant on anal pain and urinary retention after PPH surgery of damp-heat mixed hemorrhoids were evaluated based on the “Treating different diseases with the same treatment” theory combined with the “precision medicine” theory. Moreover, the mechanism of action of Prostant was investigated in animal models. We hope our results can enrich the TCM theory of “Treating different diseases with the same treatment,” as well as provide a scientific treatment method for solving anal pain and urinary retention after PPH surgery of mixed hemorrhoids.

## 2. Materials and Methods

### 2.1. Survey Object

Data of the present study were from 30 patients who were diagnosed with damp-heat mixed hemorrhoids admitted to our hospital. The diagnostic criteria were according to the “Guidelines for the Clinical Diagnosis and Treatment of Hemorrhoids” jointly stipulated by the Colorectal and Anal Surgery Group of the Chinese Medical Association Surgery Branch, the Anorectal Branch of the Chinese Society of Chinese Medicine, and the Anorectal Branch of the Chinese Society of Integrative Medicine in 2006. The diagnostic criteria for the damp-heat mixed hemorrhoids were as follows: bright red and thick blood in the stool followed by discharge or swelling of the abscess, feeling of burn pain or nourishing water, dry and loose excrement, yellow or small urine, dry mouth and tongue, yellow and dark fur, and stringy or floating pulse. A total of 30 patients with damp-heat mixed hemorrhoids were allotted into control group and treatment group (*n* = 15) through the randomized single-blind control method. Both patients in two groups obtained PPH surgery. Patients in the treatment group received Prostant before the end of the operation and placed 4 cm inside the opening of the rectum. Then, patients obtained Prostant treatment at 4 cm inside the opening of the rectum in the morning and evening after the operation for consecutive 7 days. Patients in control group did not receive any treatment. All subjects accredited the written informed consent. The study was approved by the Board and Ethics Committee of our Hospital.

#### 2.1.1. Inclusion Criteria

① Patients were in accord with the diagnostic criteria for hemorrhoids in the “Guidelines for the Clinical Diagnosis and Treatment of Hemorrhoids,” and meet the diagnostic criteria for damp-heat mixed hemorrhoids in TCM syndrome; ② The gender of the patients is not limited, and the age of the patients is between 18–70 years old; ③ The patients have obvious surgical indications and no surgical contraindications; and ④ The subjects were approved by the ethics committee of Gaozhou Hospital of Traditional Chinese Medicine affiliated to Guangzhou University of Chinese Medicine and the subjects' informed consent was obtained.

#### 2.1.2. Exclusion Criteria

① Patients were with end-stage malignant tumors or cachexia; ② Patients participated or were participating in other clinical studies during the three-month period of this study; ③ Patients were of childbearing age and have a pregnancy requirement within 3 months; ④ Patients were breastfeeding or pregnant; ⑤ Patients were with severe liver and kidney problems and malignant primary diseases such as cardiovascular and cerebrovascular defects, hematopoietic system defects, lung, and nervous system defects; ⑥ Patients were with a history of mental illness; ⑦ Patients were with inflammatory bowel disease or acute diarrhea in the past week; ⑧ Patients were with a history of urethral injury; and ⑨ Patients had other diseases that were not suitable for the present clinical trials evaluated by the investigators.

### 2.2. Diagnostic Criteria of Urinary Retention

The diagnostic criteria of urinary retention were based on the modern medical standards related to postoperative urinary retention and the diagnostic criteria of TCM obstruction.

#### 2.2.1. The Diagnostic Criteria of TCM Obstruction

The diagnostic criteria of TCM obstruction were based on the diagnostic criteria of Guiding Principles for Clinical Research of New Chinese Medicines for Treatment of Obstruction in the “Guiding Principles for Clinical Research of New Chinese Medicines” formulated and issued by the Ministry of Health of the People's Republic of China in 1993. The details were as follows:

① The patient frequently wanted to urinate, and the urine was difficult to discharge or even harder to come out at all;
② The lower abdomen accumulated more urine and felt pain;
③ It was difficult to urinate, but there was no feeling of sore urethra;
④ It was detected that there was more urine left in the bladder.

#### 2.2.2. The Diagnostic Criteria of Postoperative Urinary Retention in Modern Medicine

The diagnostic criteria of postoperative urinary retention in modern medicine were based on the clinical manifestations and diagnostic criteria of postoperative urinary retention in “surgical Complications.” The details were as follows:

① Patients did not urinate 6–8 hours after surgery;
② Patient had the following symptoms: desire to urinate, soreness, no pressure, urgency;
③ Clinical examination found bulging above the pubic joint, sore pressure, muddy sound on percussion, and palpable swollen bladder.

### 2.3. Scoring Criteria for Evaluation of Symptoms of Urinary Retention

The scoring criteria were based on the scoring criteria of “Guiding Principles for Clinical Research of New Chinese Medicines for Treatment of Obstruction.” The details were as follows:

#### 2.3.1. Urination Status

0 point, normal; 1 point, urine was as thin as a thread; 2 points, intermittent urine flow could still be thin; 3 points, urinating dripped companies with failing to form a urinary line.

#### 2.3.2. Lower Abdomen Symptoms

0 point, asymptomatic; 1 point, full of stuffiness in the lower abdomen; 2 points, discomfort in the lower abdomen; 3 points, abdominal distension with pain.

#### 2.3.3. Time to Urinate

0 point, urination time was less than 40 seconds; 1 point, urination time was between 40 and 50 seconds; 2 points, urination time was between 51 and 60 seconds; 3 points, urination time was greater than 60 seconds.

### 2.4. Scoring Criteria for Pain Assessment

Pain assessment criteria were based on the visual analog scale (VAS) (0–10 points) to directly record the score with 0 point indicating no pain and 10 points indicating maximal pain.

### 2.5. Evaluation Index

The degree of dysuria and pain of patients in the two groups was scored and analyzed. Three times were recorded at 3 h, 6 h, and 12 h after operation. After that, it was recorded again every morning and evening for 7 days. If the patient was discharged early, follow-up records would be given by telephone. In addition, the serum level of TNF-*α* and IL-6 was also measured through the enzyme-linked immunosorbent assay (ELISA) assay.

### 2.6. Animals

SPF male BALB/C mice (18–22 g) were obtained from Guangdong Medical Experimental Animal Center. All mice were bred in the SPF environment of the Animal Experiment Center of our hospital and acclimated for 3 days before experiments. Mice were offered a 12 hour/12 hour light-dark cycle and provided with water ad libitum and a standard diet at (21 ± 2)°C. All experiments were authorized by the Animal Ethics Committee of our hospital. The experimental operations and materials were conducted following the Guiding Opinions on the Treatment of Experimental Animals, Laboratory Animal Management Regulations, and Laboratory Animal Licensing Management Measures, and were consistent with the guidelines of the National Institutes of Health and Health.

### 2.7. Animals Models and Drug Administration

#### 2.7.1. Construction of a Mouse Model of Hemorrhoids

After perianal disinfection of the mice, 0.05 mL of 75% glacial acetic acid was injected into the skin around the anus. Ulcers were formed after 24 hours. Then, 2–3 mice were randomly selected and scored the perianal area based on the local symptom scoring criteria (Table 1) to evaluate the success of the model (More than 4 points indicated successful model building). In addition, hemorrhoids model was also assessed by the pathological changes of the perianal ulcer tissues.

#### 2.7.2. Construction of a Mouse Model of Hemorrhoids Combined with Urinary Retention

Mice were intraperitoneally received with 40 mg/kg sodium pentobarbital. The mouse lies in the supine position on the dissection table with a hole at the bottom. After anesthesia, the bladder is opened from the middle, and the indwelling needle is punctured and used for bladder perfusion. One end of the indwelling needle is connected to the 50 mL syringe on the micro syringe pump through a three-way valve, and the other end is connected to an amplifier through a pressure sensor. Then, the urodynamic indexes including the urination interval, maximum urination pressure, basal bladder pressure, and urination threshold pressure were recorded via the LabChart software in real-time. Next, the bladder was perfused with saline at 6 mL/h for 60 minutes to determine the urodynamic data of the bladder under normal conditions. After the last urination, the external urethra was ligated to result in obstruction and perfused with saline at 12 mL/h for two urination cycles. The acute urinary retention model was built 2 hours later.

Mice were randomly divided into control group, hemorrhoids combined with urinary retention model group (H&UR model group), hemorrhoids combined with urinary retention model treated with low-concentration of Prostant (H&UR + Prostant-low), and hemorrhoids combined with urinary retention model treated with high-concentration of Prostant (H&UR + Prostant-high) (*n* = 6). H&UR mice models were established as described above, while mice in control group obtained no stimulation. Mice in H&UR + Prostant-low and H&UR + Prostant-high groups were administrated with 0.5 g/kg and 1.0 g/kg Prostant, respectively. Therapeutic effect indexes were recorded and analyzed, including the basal pressure of the bladder during urinary retention, the time of first bladder contraction urination, the base pressure of the bladder after the first contractile urination, urination pressure, and urination threshold pressure for the first contraction of bladder urination, the base pressure of the bladder within 30 minutes after the first bladder contractile urination, urination pressure and urination threshold pressure within 30 minutes after the first bladder contraction, urination pressure during the first contraction of bladder urination, urination pressure 30 minutes after the first urinary bladder contraction and the number of unstable contractions after urination. In addition, the expression level of serum inflammatory factors, the function of T cell, the protein expression level, and the expression of VEGF in mucosa were determined by ELISA, flow cytometry, western blot, and immunohistochemistry assays.

#### 2.7.3. Construction of a Mouse Model of Hemorrhoids Combined with Urinary Retention and Pain

*(1) A mouse Model of hemorrhoids Combined with Urinary Retention and Pain was Induced by Glacial Acetic Acid*. Mice were randomly divided into control group, H&UR&P-GAA model group, H&UR&P-GAA + Prostant-low group (0.5 g/kg), and H&UR&P-GAA + Prostant-high group (1.0 g/kg) (*n* = 6). 0.6% glacial acetic acid was injected into the abdominal cavity of the hemorrhoids combined with urinary retention model mice, and then mice received chemical stimulation for 15 minutes to obtain the hemorrhoids combined with urinary retention and pain (H&UR&P-GAA) model mice. After Prostant was placed into the rectum for 20 minutes, the number of writhing reactions in the mice was recorded to indirectly evaluate the analgesic effect of Prostant.

*(2) A Mouse Model of Hemorrhoids Combined with Urinary Retention and Pain was Induced by Pressure*. Mice were randomly divided into control group, H&UR&P-pressure model group, H&UR&P-pressure + Prostant-low group (0.5 g/kg), and H&UR&P-pressure + Prostant-high group (1.0 g/kg) (*n* = 6). The tails of hemorrhoids combined with urinary retention model mice were depilated, and then the mice were placed in a fixed tube with the tail exposed. The tails were marked at 1 cm from the base of the tail, and the tail retraction was indicated as the pain response gradually increased the pressure. The pain response of mice at 0.5 h, 1 h, and 2 h after administration was measured 5 times in total, and the analgesic effect was evaluated by calculating the percentage increase in pain threshold.

*(3) A Mouse Model of Hemorrhoids Combined with Urinary Retention and Pain was Induced by fresh Egg Whites*. Mice were randomly divided into control group, H&UR&P-egg whites model group, H&UR&P-egg whites + Prostant-low group (0.5 g/kg) and H&UR&P-egg whites + Prostant-high group (1.0 g/kg) (*n* = 6). A mark was made on the right hind foot joint of the mouse, and 0.1 mL of 1% fresh egg white was injected into the foot plantar aponeurosis to cause inflammation. After 30 min, Prostant intervention was carried out and measured at 1, 2, 3, 4, and 5 hours after inflammation. The volume of the feet, the degree of swelling, and the inhibition rate of inflammation were calculated.

In addition, cytokines secreted by Th cells in serum including IL-4, IL-10, IL-17A, and IFN-*γ*, and transcriptional expression level of T-bet, ROR*γ*t, GATA3, and Foxp3 was detected by ELISA and reverse transcription-quantitative polymerase chain reaction (RT-qPCR), severally.

### 2.8. ELISA

The human serum levels of TNF-*α* and IL-6 were measured via Human IL-6 ELISA Kit (EK0410) and Human TNF*α* ELISA Kit (EK0525), and the mouse serum levels of IL-2, TNF-*α*, hs-CRP, IFN-*γ*, IL-4, IL-17A, and IL-10 were determined by Mouse IL-2 ELISA Kit (EK0398), Mouse TNF*α* ELISA Kit (EK0527), Mouse IFN gamma/IFNG ELISA Kit (EK0375), Mouse IL-4 ELISA Kit (EK0405), Mouse IL-17A ELISA Kit (EK0431), and Mouse IL-10 ELISA Kit (EK0417, BOSTER, Wuhan, China) in line with the manufacturer's protocol. The mouse serum levels of hs-CRP were analyzed through Mouse C Reactive Protein ELISA Kit (ab222511, Abcam, Cambridge, UK) based on the working instructions. The absorbance at 450 nm was measured using a microplate reader (SpectraMAX Plus384, America).

### 2.9. Flow Cytometry Assay

Mouse peripheral blood was collected and prepared into lymph node single cell suspension for flow cytometry assay. Cells were harvested and stained with anti-CD4 antibodies (all from BD Biosciences, USA). FoxP3 staining was performed using Foxp3/Transcription Factor Fixation/Permeabilization Concentrate and Diluent Kit (eBioscience, USA) according to manufacturer's instruction. Then, cells were stained with anti-FoxP3 antibody (eBioscience). Regarding intracellular cytokine staining, spleen cells were cultured in media containing PMA (50 ng/ml) and Ionomycin (1 *μ*g/ml) plus protein transport inhibitor (BD Biosciences) for 5 hours. Cells were then fixed, permeabilized, and stained with anti-IL-17A (all from eBioscience, USA). Finally, cells were analyzed using a flow cytometer (FACSCalibur, BD Biosciences). Fluorescence minus one (FMO) samples and unstained samples were used as internal controls to set positive and negative gating. Cell gating and data collection were performed in a blinded manner.

### 2.10. Western Blot Analysis

Perianal tissues were separated and lysed using RIPA lysis buffer (BOSTER). Protein concentration was determined by a Pierce® BCA Protein Assay Kit (Thermo Fisher). Subsequently, protein samples were dissolved using 10% SDS-PAGE and then transferred onto PVDF membranes (EMD Millipore, Billerica, MA, USA). The membranes were sealed in 5% skimmed milk powder at room temperature for 2 h and then hatched with primary antibodies overnight at 4°C, followed by introduction with the corresponding secondary antibody (BOSTER) for 1 h at room temperature. An ECL chemiluminescence kit (EMD Millipore) was used to visualize the bands, and Image-ProPlus software (Media Cybernetics, Inc., Rockville, MD, USA) was utilized for the analysis of the gray value. Protein levels were calculated relative to GAPDH. The primary antibodies used in this study were as follows: GAPDH (ab181602, Abcam), JAK2 (74987, Cell Signaling Technology, Inc., Danvers, MA, USA), and phospho-STAT3 (Tyr705) (9145, CST) at 1 : 1, 000 dilutions.

### 2.11. Immunohistochemistry (IHC)

Internal hemorrhoid-damaged mucosal tissues were immobilized with 4% paraformaldehyde *f* at room temperature for 24 h. After dehydrated, embedded, and cut, the sections (5 *μ*m) were obtained for IHC experiment. Sections were stained with anti-VEGFC antibody (A00623, BOSTER). Subsequently, the slices were assessed with a light microscope (Olympus, Tokyo, Japan) and Image-Pro Plus 6.0 (Media Cybernetics).

### 2.12. RT-qPCR Analysis

Total RNA from perianal tissues was enriched through TRIzol reagent (TaKaRa Biotechnology Co., Ltd., Dalian, China) and then reversely transcribed into cDNA by PrimeScript™ RT reagent Kit with gDNA Eraser (Perfect Real Time) (TaKaRa) following the operating instruction. RT-qPCR was performed in a 25 *μ*l quantum involving 2 *μ*l of the cDNA, 12.5 *μ*l 2 × SYBR® Premix Ex Taq™ II (Tli RNaseH Plus) (TaKaRa), 2 *μ*l of the 10-*μ*M upstream and downstream primers as well as 8.5 *μ*l ddH_2_O, using the ABI PrismTM 7500 (Thermo Fisher). The RT-qPCR conditions were as follows: 30 s at 95°C, followed by 40 cycles between 95°C for 5 s and 60°C for 30 s, 95°C for 10 s, and 65°C for 5 s. The relative expressions of T-bet, ROR*γ*t, GATA3, and Foxp3 were calculated using the 2^−ΔΔCT^ method and normalized to the GAPDH. All the primers used in the present study were listed in Table 2.

### 2.13. Statistical Analysis

Data in the present study were determined using SPSS 25.0 statistical software (IBM, Armonk, New York, USA). Data were first analyzed using Shapiro‒Wilk test. All the measurement data were presented as means ± SD when measurement data conformed to normal distribution. Data were tested by a rank sum test or student's *t*-test with only two groups, whereas differences were determined via one-way analysis of variance and Duncan's test among various groups. All the count data were displayed as a percentage and detected by the chi-square test. The differences were regarded as statistically significant when *p* < 0.05. Figures were generated using Graphpad Prism 8.0 software.

## 3. Results

### 3.1. General Information

30 patients who accorded with the inclusion and exclusion criteria were included and allocated into control and treatment groups. As shown in Table 3, 15 patients contained 9 males and 6 females aged 23–69 years (41.0 ± 13.2 years) with medical history 0.5–10 years (4.1 ± 3.1 years) in control group. In the treatment group, 15 patients included 10 males and 5 females aged 24–69 years (45.5 ± 15.1 years) with medical history 1–30 years (7.3 ± 7.9 years). Data of the two groups showed no statistical difference (*p* > 0.05).

### 3.2. The Clinical Effect of Prostant on Urinary Retention and Pain

**Figure 1:**
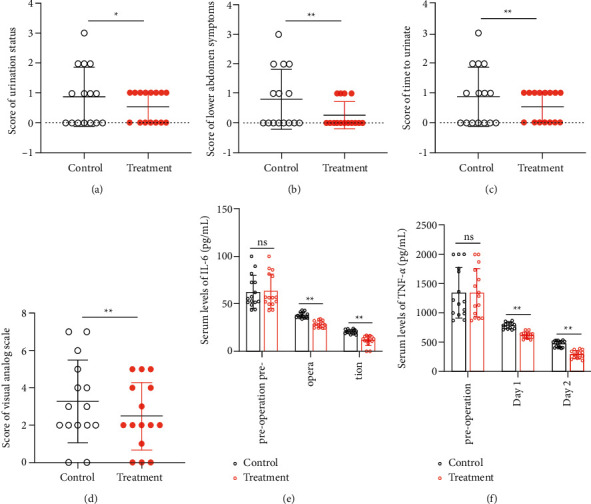
Prostant effectively alleviated urinary retention, pain, and inflammation. The symptoms of urinary retention including urination status (a), lower abdomen symptoms (b) and time to urinate (c) were scored, and the score of VAS (d) was also quantified to evaluate the pain. In addition, the serum level of IL-6 (e) and TNF-*α* (f) was determined by ELISA before operation and on the first day, and the second day after the operation. Data in (a)–(d) were evaluated by the rank sum test, while data in (e) and (f) were examined by the student's *t*-test. The means ± SD of 15 independent samples were presented. ^*∗*^*p* < 0.05 and  ^*∗∗*^*p* < 0.01.

Patients with the damp-heat mixed hemorrhoids received PPH surgery and then treated with or without Prostant. Clinical data revealed that Prostant treatment significantly ameliorated the symptoms of urinary retention including urination status (Figure 1(a)), lower abdomen symptoms (Figure 1(b)), and time to urinate (Figure 1(c)). In addition, patients treated with Prostant notably suffered less pain as indicated by a prominently lower score of VAS (Figure 1(d)). Moreover, ELISA results exhibited that the serum level of IL-6 and TNF-*α* of the two groups was no statistical difference before operation, while IL-6 and TNF-*α* levels in patients administrated with Prostant were observably reduced on the first day and the second day after the operation (Figures 1(e) and 1(f)). In summary, these outcomes indicated that Prostant could effectively relieve urinary retention, pain, and inflammation.

### 3.3. Prostant Ameliorated the Urinary Retention in Mice Model

**Figure 2:**
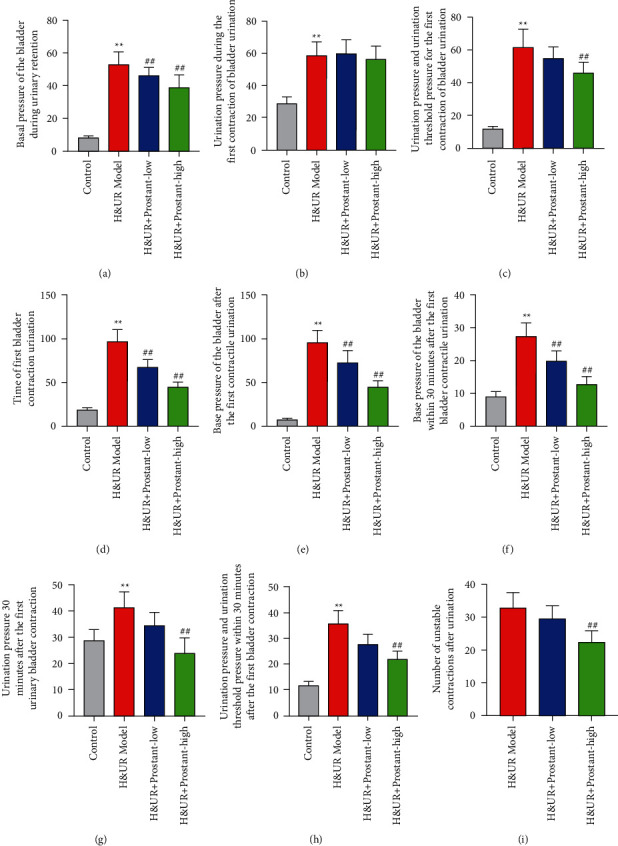
Prostant improved the urinary retention in mice model. Mice with hemorrhoids combined with urinary retention were treated with or without Prostant. Urodynamics, including the basal pressure of the bladder during urinary retention (a), the urination pressure during the first contraction of bladder urination (b), the urination pressure and urination threshold pressure for the first contraction of bladder urination (c), the time of first bladder contraction urination (d), the base pressure of the bladder after the first contractile urination (e), the base pressure of the bladder within 30 minutes after the first bladder contractile urination (f), the urination pressure 30 minutes after the first urinary bladder contraction (g), the urination pressure and urination threshold pressure within 30 minutes after the first bladder contraction (h), and the number of unstable contractions after urination (i). The means ± SD of six independent samples were shown.  ^*∗∗*^*p* < 0.01 v.s control group. ^##^*p* < 0.01 v.s H&UR model group.

To reveal the underlying mechanism of the effect of Prostant on urinary retention and pain, we built a mice model of hemorrhoids combined with urinary retention. Urodynamics results revealed that both low-dose and high-lose Prostant significantly attenuated the H&UR-induced enhancement of the basal pressure of the bladder during urinary retention (Figure 2(a)), the time of first bladder contraction urination (Figure 2(d)), the base pressure of the bladder after the first contractile urination (Figure 2(e)), and the base pressure of the bladder within 30 minutes after the first bladder contractile urination (Figure 2(f)). However, only high-lose Prostant prominently declined the H&UR-induced increase of the urination pressure and urination threshold pressure for the first contraction of bladder urination (Figure 2(c)), urination pressure 30 minutes after the first urinary bladder contraction (Figure 2(g)), urination pressure and urination threshold pressure within 30 minutes after the first bladder contraction (Figure 2(h)), and the number of unstable contractions after urination (Figure 2(i)). Besides, both low-dose and high-lose Prostant had no effect on the H&UR-induced augmentation of the urination pressure during the first contraction of bladder urination (Figure 2(b)). In brief, these data suggested that Prostant could improve urinary retention in the mice model.

### 3.4. Prostant Regulated Inflammatory Factors Expression through Modulating Th17/Treg Balance with Inhibiting JAK/STAT Signaling Pathway

**Figure 3:**
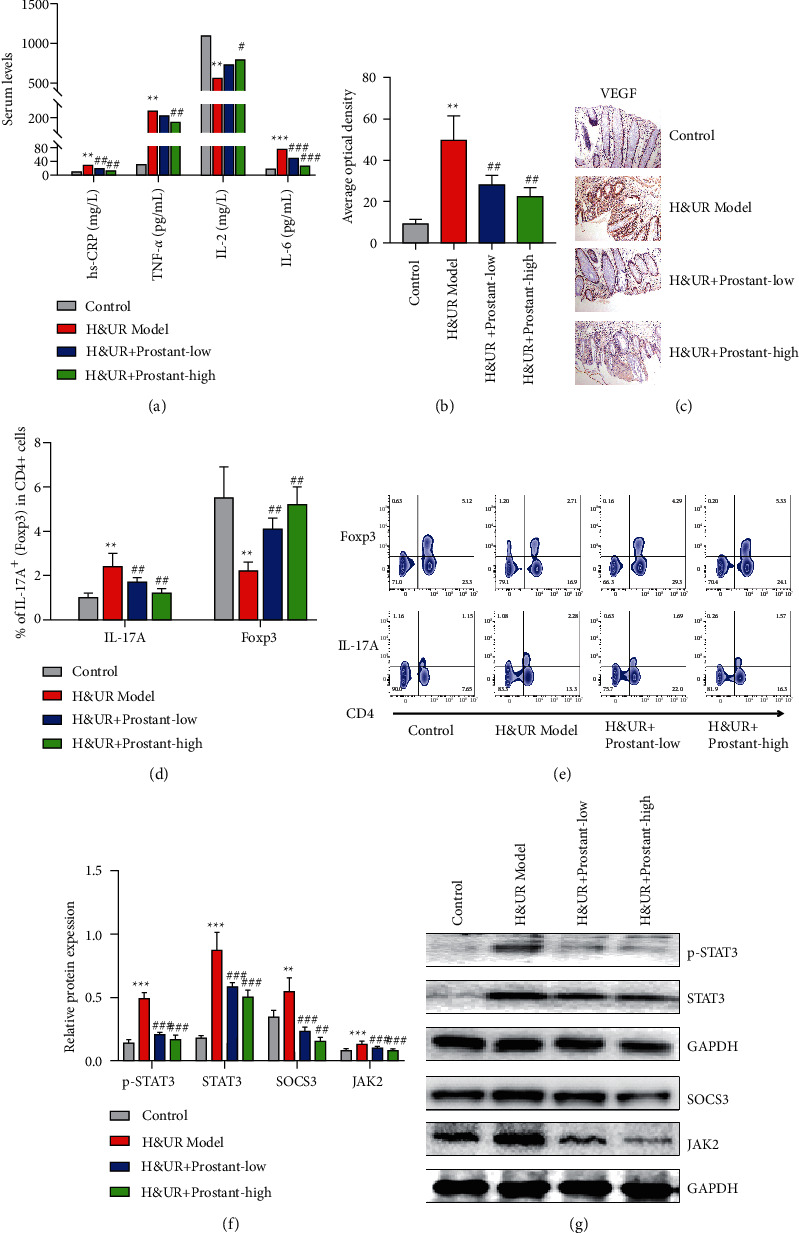
Prostant regulated inflammatory factors expression through modulating Th17/Treg balance with inhibiting JAK/STAT axis. (a) The serum level of hs-CRP, TNF-*α*, IL-2, and IL-6 was examined via ELISA. ((b) and (c)) The level of VEGF in mucosal tissues was determined by IHC. ((d) and (e)) The expression of Th17 and Treg was assessed by flow cytometry assay. ((f) and (g)) The protein expression of STAT3, phosphorylated STAT3, SOCS3, and JAK2 was evaluated by western blot. Data were exhibited following being normalized to GAPDH. The means ± SD of six independent samples were displayed.  ^*∗∗*^*p* < 0.01 and  ^*∗∗∗*^*p* < 0.001 v.s control group. ^#^*p* < 0.05, ^##^*p* < 0.01 and ^###^*p* < 0.001 v.s H&UR model group.

The serum level of inflammatory factors including hs-CRP, IL-6, and TNF-*α* was notably increased in H&UR model mice, which was markedly reversed with high-dose Prostant treatment (Figure 3(a)). On the contrary, high-dose Prostant introduction notably promoted the H&UR-induced decrease of the serum level of IL-2 (Figure 3(a)). IHC analysis revealed that the H&UR-induced elevation of VEGF expression was dramatically reduced by both low-dose and high-dose Prostant treatment (Figures 3(b) and 3(c)). Additionally, flow cytometry results revealed that both low-dose and high-dose Prostant prominently downregulated the H&UR-induced increase of Th17 expression, but upregulated the H&UR-induced decrease of Treg expression (Figures 3(d) and 3(e)). The relative protein levels of JAK2, STAT3, and p-STAT3 were notably enhanced in H&UR model mice, which was observably antagonized with both low-dose and high-dose Prostant treatment (Figures 3(f) and 3(g)). Based on these results, we demonstrated that Prostant regulated Th17/Treg balance in urinary retention mice by suppressing the expression of JAK/STAT axis, thereby modulating the expression of inflammatory factors, and finally improving urinary retention.

### 3.5. Prostant Exerted Analgesic Effect on Mice

**Figure 4:**
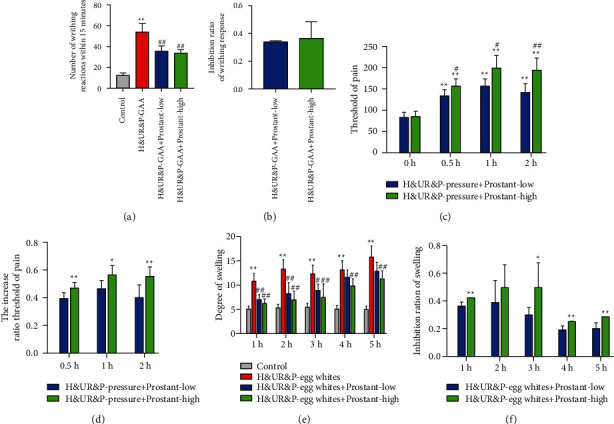
Prostant exerted analgesic effect on mice. H&UR model mice were introduced with 0.6% glacial acetic acid, and then the number of writhing reactions (a) and the inhibition ratio of writhing response (b) were detected. In addition, H&UR model mice were pressed and analyzed at 0.5 h, 1h, and 2h after administration. The threshold of pain (c) and the increased ratio threshold of pain (d) were determined. Besides, the foot plantar aponeurosis of H&UR model mice was injected with 1% fresh egg white to cause inflammation. The degree of swelling (e) and the inhibition rate of inflammation (f) were assessed. The means ± SD of six independent samples were displayed. In Figures (a) and (e),  ^*∗∗*^*p* < 0.01 v.s control group, and ^##^*p* < 0.01 v.s H&UR model group. In Figure (c),  ^*∗∗*^*p* < 0.01 v.s that at 0 h, ^#^*p* < 0.05 and ^##^*p* < 0.01 v.s low-dose prostant at the same time point, respectively. In Figures (d) and (f), ^*∗*^*p* < 0.05 and  ^*∗∗*^*p* < 0.01 v.s low-dose prostant at the same time point, respectively.

Next, three pain mice models were built to assess the effect of Prostant on the pain. After H&UR model mice were administrated with 0.6% glacial acetic acid, the number of writhing reactions was significantly increased, which was notably inverted with both low-dose and high-dose Prostant treatment (Figure 4(a)). Thus, the inhibition ratio of writhing response between low-dose and high-dose Prostant treatment was no statistical difference (Figure 4(b)). Besides, H&UR model mice were pressed and analyzed at 0.5 h, 1 h, and 2 h after administration. The results revealed that both low-dose and high-dose Prostant treatment prominently elevated the threshold of pain of H&UR model mice at 0.5 h, 1 h, and 2 h compared with that at 0 h (Figure 4(c)). Moreover, high-dose Prostant treatment further significantly increased the threshold of pain of H&UR model mice compared with low-dose Prostant treatment at 0.5 h, 1 h, and 2 h (Figure 4(c)). Therefore, high-dose Prostant treatment observably enhanced the increased ratio threshold of pain compared with low-dose Prostant treatment (Figure 4(d)). Similarly, the foot plantar aponeurosis of H&UR model mice was injected with 1% fresh egg white to cause inflammation. The degree of swelling of H&UR model mice was memorably enhanced relative to that in control mice at 1, 2, 3, 4, and 5 h after inflammation. However, both low-dose and high-dose Prostant treatment notably attenuated the degree of swelling of H&UR model mice at 1, 2, and 3 h, while only high-dose Prostant treatment prominently reduced the degree of swelling of H&UR model mice at 4 and 5 h (Figure 4(e)). Hence, high-dose Prostant treatment significantly elevated the inhibition rate of inflammation compared with low-dose Prostant treatment at 1, 2, 3, 4, and 5 h (Figure 4(f)). Altogether, we concluded that Prostant had an analgesic effect on mice.

### 3.6. Prostant Exerted Analgesic Effect via Regulating the Balance between T Cell Subpopulations

**Figure 5:**
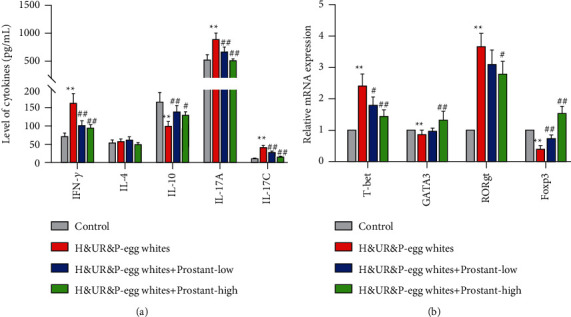
Prostant exerted analgesic effect via regulating the balance between T cell subpopulations. (a) The serum level of IFN-*γ*, IL-4, IL-10, IL-17A, and IL-17C was detected by ELISA. (b) The mRNA expression level of T-bet, ROR*γ*t, GATA3, and Foxp3 was measured by qRT-PCR. Data were exhibited following being normalized to GAPDH. The means ± SD of six independent samples were displayed.  ^*∗∗*^*p* < 0.01 and  ^*∗∗∗*^*p* < 0.001 v.s control group. ^#^*p* < 0.05, ^##^*p* < 0.01 and ^###^*p* < 0.001 v.s H&UR&P model group.

Based on the above-mentioned results, the balance between T cell subpopulations may exert a crucial role in urinary retention and pain. Thus, the serum level of IFN-*γ*, IL-4, IL-10, IL-17A, and IL-17C was detected. ELISA results exhibited that both low-dose and high-dose Prostant treatment notably reduced H&UR&P-induced elevation of the serum level of IFN-*γ*, IL-17A, and IL-17C, while both low-dose and high-dose Prostant treatment observably enhanced H&UR&P-induced reduction of the serum level of IL-10 (Figure 5(a)). However, the serum level of IL-4 was no statistical difference among the four groups (Figure 5(a)). Furthermore, qRT-PCR results uncovered that the transcriptional level of T-bet and ROR*γ*t was observably increased, which was significantly reversed with both low-dose and high-dose Prostant treatment (Figure 5(b)). The mRNA expression level of GATA3 and Foxp3 was markedly reduced, which was notably rescued with high-dose Prostant treatment (Figure 5(b)). Therefore, these data indicated that Prostant mainly exerted an analgesic effect via modulating the balance of Th17/Treg.

## 4. Discussion

In the current study, we explored the clinical effects of as well as the potential molecular mechanism of Prostant on urinary retention and anal pain based on the “Treating different diseases with the same treatment” theory combined with “precision medicine” theory. We found that Prostant could treat patients and mice with urinary retention and anal pain. Mechanically, Prostant may modulate the balance of Th17/Treg to regulate the secretion and production of inflammatory factors, which ameliorated urinary retention and anal pain.

Prostant has been demonstrated to be effective and safe in the treatment of chronic prostatitis [15]. The pathological mechanism of chronic prostatitis according to TCM theories is damp-heat stasis, which is similar to that of urinary retention after PPH surgery of damp-heat mixed hemorrhoids. Thus, Prostant was used to treat the patients with urinary retention after PPH surgery in the present study based on the “treating different diseases with the same treatment” theory. Satisfactorily, Prostant was also effective for the patients with urinary retention after PPH surgery as indicated by the improvement of urination status, lower abdomen symptoms, and time to urinate. During the surgery, the traction, sharp cutting, or thermal damage around the anorectum and perineum can damage the muscles, blood vessels, and nerves, which results in the release of inflammatory factors, local inflammation, pain, local bleeding, and hematoma formation. These stimuli can reflexively cause muscle spasms and wound inflammation in the perineum, prostate, and bladder neck, which can lead to dysuria and urinary retention [16]. Thus, a previous study has highlighted that the risk of urinary retention is tightly associated with inflammation [17]. Wu et al. [18] have exhibited that the serum concentration of IL-6 was notably enhanced in patients with acute urinary retention. Our ELISA results revealed that Prostant treatment notably diminished the serum level of proinflammatory cytokines including TNF-*α* and IL-6 both on the first and second day after the operation. TNF-*α*, a core modulator of inflammatory responses, has been identified to be relevant to the pathogenesis of inflammatory [19]. IL-6 exerts a significant role in a variety of signs of progress, such as inflammation, immune regulation, hematopoiesis, and oncogenesis [20]. In addition, the degree of pain was also markedly alleviated with Prostant treatment as shown by the reduction of score of VAS. Therefore, we concluded that Prostant might attenuate the secretion of inflammatory mediators to exert anti-inflammatory function, which slowed down the course of the disease, thereby reducing the pain after the operation.

Then, a urinary retention model was built, and urodynamics results revealed that Prostant treatment observably improved urinary retention in mice. CRP is an acute phase protein secreted by IL-6 that is upregulated during various inflammatory processes, tissue necrosis, and tissue damage (such as after surgery) [21]. Thus, serum CRP has been demonstrated to be a predictor due to an increase of its serum level in acute urinary retention, [22] acute prostatitis [23], and liver cirrhosis [24]. IL-2 regulates the cellular activity of white blood cells in the immune system [25]. Similarly, ELISA results revealed that Prostant administration significantly declined the H&UR-induced enhancement of the serum level of hs-CRP and TNF-*α*, while enhancing the H&UR-induced reduction of the serum level of IL-2. It has been elucidated that the balance of Treg/Th17 is correlative with inflammation in various diseases, such as colon inflammation, [26] experimental autoimmune encephalomyelitis, [27] rheumatoid arthritis [28], and chronic obstructive pulmonary disease [29]. In line with these previous studies, our results also showed the unbalance of Treg/Th17 in the mice with urinary retention. However, Prostant treatment prominently rescued the dysregulation of Treg/Th17. Furthermore, Prostant treatment observably antagonized the H&UR-induced enhancement of JAK2 and phosphorylated STAT3. JAK/STAT signal pathway is a core pathway for intracellular signal transduction, which has been introduced to be essential for the differentiation of CD4^+^ T cells into effector or regulatory phenotypes [30, 31]. Taken together, we concluded that Prostant can modulate Th17/Treg balance in urinary retention mice by inhibiting JAK/STAT signaling pathway, which regulates the generation of inflammatory cytokines to improve urinary retention.

In addition, the balance of Th17/Treg has also been demonstrated to be associated with pain, such as neuropathic pain, [32] bone cancer pain [33], and chronic low back pain [34]. Similarly, serum level of IL-17A was notably elevated in H&UR&P mice model, which could be reversed with Prostant treatment. Furthermore, Prostant treatment prominently antagonized the H&UR&P-induced increase of ROR *γ*t, and reduction of FoxP3. ROR *γ*t is a pivotal transcription factor for Th17 cells differentiation, and it also regulates the expression and secretion of IL-17, [35] while FoxP3 is a representative transcription factor of Treg cells [36]. Moreover, Th1 cells can promote the occurrence of pain according to the production of proinflammatory cytokines TNF and IFN-*γ*, while Th2 cells can relieve pain based on the generation of anti-inflammatory cytokines IL-4, IL-5, IL-10, and IL-13 [37]. T-bet and GATA-3 can mediate the differentiation of T cells into Th1 and Th2, respectively [38]. Thus, in the present study, Prostant treatment prominently antagonized the H&UR&P-induced increase of IFN-*γ* and T-bet, and reduction of IL-10 and GATA-3. In brief, combined with the improvement of pain with Prostant treatment as indicated by the reduction of writhing response, and the elevation of threshold of pain and degree of swelling, we concluded that Prostant can alleviate urinary retention and pain through regulating the balance between T cell subpopulations in mice. Nevertheless, no statistical difference was detected in the serum level of IL-4 relative to that in the control, which might suggest that Prostant mainly alleviated urinary retention and pain through regulating the balance of Th17/Treg.

In conclusion, Prostant can treat patients and mice with anal pain and urinary retention. Mechanically, Prostant may regulate the balance of Th17/Treg to modulate the secretion and production of inflammatory factors, which ameliorated anal pain and urinary retention. However, our study still has a few limitations. First, the clinical sample size of this study is small, thus more sample numbers are needed in subsequent observations. Next, the potential and further molecular mechanisms (such as some relevant signaling pathways) involved in pain need to be inquired about in further study. Additionally, the lack of validation of jak2/stat3 inhibitors and/or agonists is indeed another shortcoming of our study, which need to be supplemented in the following experiments. Briefly, we hope the results can establish a foundation for therapeutic drugs to treat anal pain and urinary retention after surgery.

## Figures and Tables

**Table 1:** Local symptoms scale.

Scores	0	1	2	3
Degree of swelling	None	Barely visible	Edge higher than skin	Markedly swollen
Ulcer area	None	＜0.2 cm^2^	＜0.3 cm^2^	＞0.3 cm^2^

**Table 2:** Primer sequences included in the present study.

Primer name	Sequence (5′–3′)
GAPDH-F	GGAGAGTGTTTCCTCGTCCC
GAPDH-R	ACTGTGCCGTTGAATTTGCC
T-bet F	AAGTTCAACCAGCACCAGACAGAG
T-bet R	GCCACGGTGAAGGACAGGAATG
ROR*γ*t F	AAGGCAAATACGGTGGTGTG
ROR*γ*t R	GAAAAGGGTGAAGGAGTCGC
GATA3 F	CCAAGGCACGATCCAGCACAG
GATA3 R	TTATGGTAGAGTCCGCAGGCATTG
Foxp3 F	AAGAATGCCATCCGCCACAACC
Foxp3 R	GGCGTTGGCTCCTCTTCTTGC

**Table 3:** Comparison of general information between control group and treatment group.

	Control (*n*)	Treatment (*n*)	*χ * ^2^	*p*
Gender				
Male	9	10	0.000	1.000
Female	6	5		

Age				
＜35(year)	6	6	3.333	0.343
35–50(year)	4	3		
＞50(year)	5	6		

Medical history				
＜3.0(year)	5	4	0.000	1.000
3.0–5.0(year)	6	5		
＞5.0(year)	4	6		

## Data Availability

The data analyzed and used during the present study are available from the corresponding author on reasonable request.
